# Identification of a novel linear B-cell epitope in the UL26 and UL26.5 proteins of Duck Enteritis Virus

**DOI:** 10.1186/1743-422X-7-223

**Published:** 2010-09-13

**Authors:** Xiaoli Liu, Zongxi Han, Yuhao Shao, Dan Yu, Huixin Li, Yu Wang, Xiangang Kong, Shengwang Liu

**Affiliations:** 1 Division of Avian Infectious Diseases, National Key Laboratory of Veterinary Biotechnology, Harbin Veterinary Research Institute, the Chinese Academy of Agricultural Sciences, Harbin 150001, the People's Republic of China

## Abstract

**Background:**

The Unique Long 26 (UL26) and UL26.5 proteins of herpes simplex virus are known to function during the assembly of the viruses. However, for duck enteritis virus (DEV), which is an unassigned member of the family *Herpesviridae*, little information is available about the function of the two proteins. In this study, the C-terminus of DEV UL26 protein (designated UL26c), which contains the whole of UL26.5, was expressed, and the recombinant UL26c protein was used to immunize BALB/c mice to generate monoclonal antibodies (mAb). The mAb 1C8 was generated against DEV UL26 and UL26.5 proteins and used subsequently to map the epitope in this region. Both the mAb and its defined epitope will provide potential tools for further study of DEV.

**Results:**

A mAb (designated 1C8) was generated against the DEV UL26c protein, and a series of 17 partially overlapping fragments that spanned the DEV UL26c were expressed with GST tags. These peptides were subjected to enzyme-linked immunosorbent assay (ELISA) and western blotting analysis using mAb 1C8 to identify the epitope. A linear motif, ^520^IYYPGE^525^, which was located at the C-terminus of the DEV UL26 and UL26.5 proteins, was identified by mAb 1C8. The result of the ELISA showed that this epitope could be recognized by DEV-positive serum from mice. The ^520^IYYPGE^525 ^motif was the minimal requirement for reactivity, as demonstrated by analysis of the reactivity of 1C8 with several truncated peptides derived from the motif. Alignment and comparison of the 1C8-defined epitope sequence with those of other alphaherpesviruses indicated that the motif ^521^YYPGE^525 ^in the epitope sequence was conserved among the alphaherpesviruses.

**Conclusion:**

A mAb, 1C8, was generated against DEV UL26c and the epitope-defined minimal sequence obtained using mAb 1C8 was ^520^IYYPGE^525^. The mAb and the identified epitope may be useful for further study of the design of diagnostic reagents for DEV.

## Background

Herpesviruses exist widely in nature. The genomes of herpesviruses consist of linear double-stranded DNA; they differ in size (from approximately 124 to 235 kb), sequence arrangement and base composition [1], and vary significantly with respect to the presence and arrangement of inverted and directly repeated sequences. The genomes of most of the alphaherpesviruses, such as herpes simplex virus 2 (HSV-2) [2] and Marek's disease virus 1 (MDV-1) [3], encode more than 70 proteins; some of these proteins are not essential for the replication of the viruses. Only limited information is available about the structures and functions of these 70 proteins, although some studies of the antigenic determinants of the glycoproteins have been reported [4,5]. Three types of capsid, named A-, B-, and C-capsids, are needed in the assembly of HSV-1 [6]. B-capsids lack DNA but may be the important intermediates in virus assembly [7-10]. The unique feature of B-capsids is the presence of an abundant core protein, named scaffolding protein ICP35 (VP22a) [6,11-13], which is encoded by the in-frame gene *UL26.5*. This protein is present in the B-capsids of the HSV-1 assembly but is absent after the completion of DNA encapsidation and is not found in the mature virion [14].

Duck enteritis virus (DEV), an unassigned member of the family *Herpesviridae *[15], is the cause of duck viral enteritis (DVE), which is also known as duck plague (DP), a disease of *Anseriformes*. DVE is a form of hemorrhagic enteritis that occurs in captive or free-flying waterfowl [16] and causes heavy economic losses in commercial duck production [17]. The DEV establishes an asymptomatic carrier state in waterfowl in the course of infection, and it is only detectable during the intermittent shedding period of the infection [18]. Currently, only limited information is available on the genomic sequence and encoded proteins of DEV; therefore the development of diagnostic methods based on virus detection is difficult. Hence, the development of immunity based prophylactic, therapeutic, and diagnostic techniques for the control DEV is of significance.

The DEV has a linear double-stranded DNA genome of approximately 180 kb with a G+C content of 64.3% [16]. The genes and their arrangements in the DEV UL region have been reported by our laboratory [19-23]. Our results have demonstrated that DEV *UL26 *and *UL26.5*, two nested in-frame genes, encode a capsid maturation protease and the minor capsid scaffold protein of DEV [20].

B-cell epitopes are antigenic determinants that are recognized and bound by membrane-associated receptors on the surface of B lymphocytes [24]. They can be classified into two types: linear (continuous) epitopes and conformational (discontinuous) epitopes. Linear epitopes are short peptides that correspond to a contiguous amino acid sequence within a protein [25,26]. To date, there has been no study of the B-cell epitopes of DEV. In this study, we first expressed the 360 amino acids in the C-terminus of the DEV UL26 protein (named UL26c), which contain the whole sequence of UL26.5. Subsequently, we generated a monoclonal antibody (mAb) (named 1C8) against DEV UL26 by vaccination of mice with a recombinant UL26c prime and DEV particle boost. Finally, we identified an epitope in the DEV UL26 protein which was recognized by the mAb 1C8. These results will provide a basic understanding of the structure, function and localization of the DEV UL26 protein and UL26.5 protein. This mAb and the recombinant proteins could be used as a potential tool for the design of diagnostic reagents for DEV.

## Results

### Production of the recombinant protein UL26c

**Figure 1:**
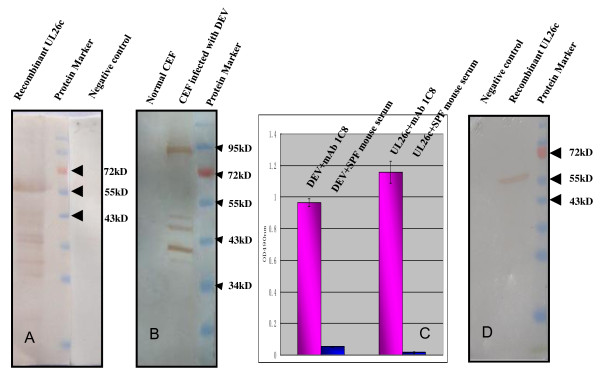
**Reactivity of mAb 1C8 with DEV and recombinant UL26c protein by western blotting and ELISA**. A) The western blotting results showed the reactivity of recombinant protein UL26c with murine anti-DEV serum; *E. coli *BL 21 (DH3) without induction was used as a negative control. B) Western blotting results showed the reactivity of CEF infected with DEV with mAb 1C8; normal CEF were used as negative controls. C) The ELISA results showed the reactivities of DEV in CEF with mAb 1C8 and the recombinant protein UL26c with mAb 1C8; SPF mouse sera were used as negative controls. D) The recombinant protein UL26c was probed with mAb 1C8. *E. coli *BL 21 (DH3) without induction was used as a negative control.

Owing to the difficulty of expressing the whole *UL26 *gene (in total 2124 bp in length) of DEV, we used prokaryotic expression of the C-terminal 360-amino acid (348-707aa) protein in *E. coli *BL21 (DE3) after induction by Isopropyl-β-D-thiogalactopyranoside (IPTG) and termed the recombinant protein UL26c. Western blotting using murine antibody against DEV showed that UL26c could react with DEV antibody (Figure 1A), which implied that it had similar antigenicity to native DEV UL26 protein. The UL26c was used to prime immunity and DEV particles were used as boost antigens to prepare the mAb.

### Generation of mAb against the DEV UL26 protein

One hybridoma clone that secreted mAb specific to the DEV UL26 protein was selected and designated as 1C8. The subclass of 1C8 was determined to be IgM with κ light chain. Both western blotting and ELISA showed that 1C8 could react specifically with both chicken embryonic fibroblasts (CEF) infected with DEV (Figure 1B and Figure 1C) and the recombinant protein UL26c (Figure 1D and Figure 1C). In summary, we can conclude that the mAb 1C8 recognized the DEV UL26 protein specifically.

### Mapping and identification of the epitope of UL26 protein

**Figure 2:**
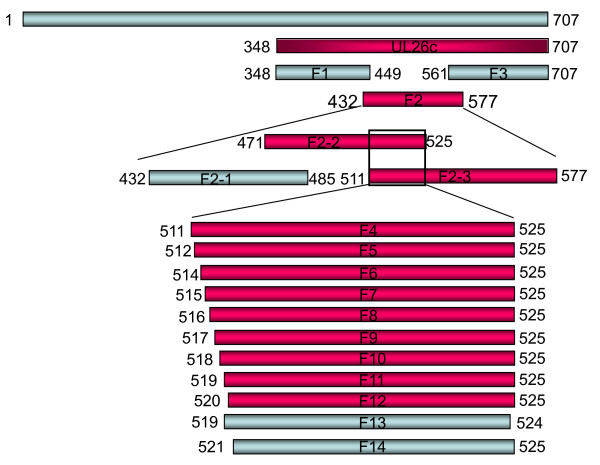
**Schematic diagram showing the truncated fragments derived from the UL26c protein of DEV Clone-03 strain and their relative positions**. Letters represent the amino acid positions in the UL26 protein. The names of the peptides are as in Table 1. The bars represent peptides of the truncated DEV UL26 proteins. The peptides that were negative in western blotting and ELISA with mAb 1C8 are shown in gray and the peptides that were positive in western blotting and ELISA with mAb 1C8 are shown in pink.

**Figure 3:**
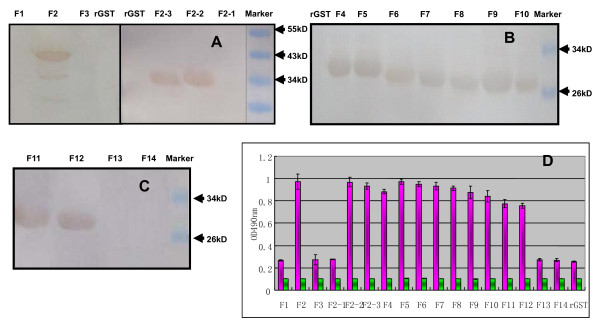
**Precise localization of the epitope defined by mAb 1C8**. The reactivity of mAb 1C8 with different truncated recombinant proteins was determined by western blotting and ELISA. The names of the proteins are the same as in Table 1. Recombinant GST (rGST) protein was used as a negative control in both the western blotting and indirect ELISA. A) The western blotting results of mAb 1C8 with peptides F1, F2, F3, F2-1, F2-2 and F2-3. B) The western blotting results of mAb 1C8 with peptides F4, F5, F6, F7, F8, F9 and F10. C) The western blotting results of mAb 1C8 with peptides F11, F12, F13 and F14. D) The results of ELISA of mAb 1C8 with the 17 recombinant proteins. The pink columns indicated the results of the ELISA of mAb 1C8 with the 17 recombinant proteins and the green columns are negative controls, which showed the results of ELISA of SP2/0 cell culture media with the recombinant proteins.

For fine mapping of the epitope of DEV UL26 that was recognized by mAb 1C8, a set of GST fused proteins (Figure 2) were expressed in prokaryotes and used together to identify the epitope by both western blotting and ELISA. Western blotting showed that the peptide F2 (432-577aa) was recognized by mAb 1C8 (Figure 3A). The further screening results showed that both F2-2 (471-525aa) and F2-3 (511-577aa) could react with mAb 1C8, while F2-1 (432-485aa) failed to be recognized (Figure 3A). These results indicated that the overlapping sequence shared by F2-2 and F2-3 may contain the epitope recognized by mAb 1C8. Hence, to define the epitope defined by mAb 1C8 in the UL26 protein more precisely, a series of truncated peptides (from F4 to F 14; Figure 2) that were deleted successively from the amino or carboxyl terminuses of the overlapping fragment were expressed, respectively, for subsequent screening of mAb 1C8. The results showed that the mAb 1C8 reacted with peptides from F4 (511-525aa) to F12 (520-525aa) (Figure 3B and Figure 3C), but not with F13 (519-524aa) and F14 (521-525aa) (Figure 3C). Therefore, we considered that the motif ^520^IYYPGE^525 ^is the defined minimal epitope in the UL26 protein of the DEV Clone-03 strain that is recognized by mAb 1C8, because deletion of I^520 ^or E^525 ^destroyed the binding of the GST fusion peptides by mAb 1C8. In addition, the results were confirmed further by ELISA (Figure 3D).

### Reaction of the epitope peptide with duck anti-DEV antibody

**Figure 4:**
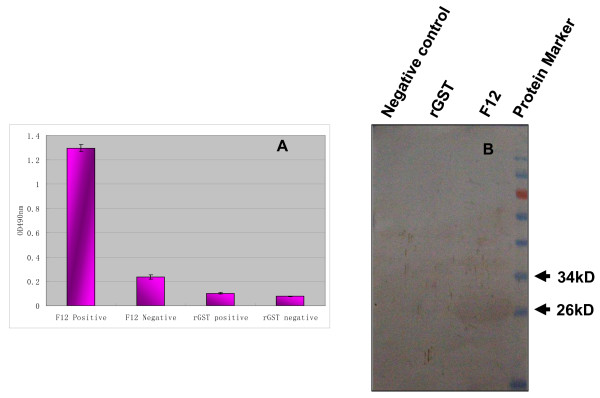
**Reactivity of the identified epitope (F12: ^520^IYYPGE^525^) with antibodies against DEV**. A) The peptide that corresponded to the epitope defined by mAb 1C8 was used as the coating antigen in an ELISA, and purified rGST protein was used as a negative control. The murine anti-DEV antibody was used as the primary antibody and SPF mouse sera were used as negative antibody controls. B) The western blotting results of the epitope defined by mAb 1C8 with the murine antibody against DEV; the rGST and *E. coli *BL 21 (DH3) without induction were used as negative controls.

The recombinant peptide, F12 (^520^IYYPGE^525^), was used as an antigen to react with mouse anti-DEV antibody in this study. The results of both western blotting and ELISA showed that this peptide defined by mAb 1C8 could react with murine anti-DEV antibody (Figure 4), which demonstrated that the epitope had good reactivity.

### Alignment of the 1C8-defined sequences in alphaherpesviruses

**Figure 5:**
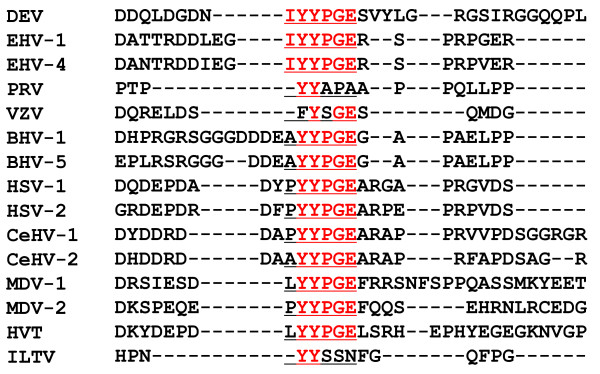
**Alignment of the sequences in the epitope motif with 14 herpesviruses in subfamily *Alphaherpesvirinae***. The epitope sequences are underlined and the amino acid residues in the epitope region that are shared by different herpesviruses are shown in red. Hyphens indicated the deleted amino acid residues. EHV: equine herpesvirus; PRV: pseudorabies virus; VZV: varicella-zoster virus; BHV: bovine herpesvirus; HSV: herpes simplex virus; CeHV: cercopithecine herpesvirus; MDV: Marek's disease virus; HVT: turkey herpesvirus; ILTV: infectious laryngotracheitis virus.

The mAb 1C8-defined motif and flanking sequences in the UL26 protein of alphaherpesviruses were aligned and analyzed. The results showed that the epitope defined by mAb 1C8, ^520^IYYPGE^525^, was relatively conserved in alphaherpesviruses (Figure 5). Interestingly, EHV-1 and EHV-4 have the same amino acid residues as DEV in the motif defined by mAb 1C8. Two residues of the epitope, ^521^YY^522^, were highly conserved among the alphaherpesviruses, except for the residue F^521 ^in VZV. In addition, the C-terminus of the amino acids of the epitope was highly conserved among most of the alphaherpesviruses selected for this study (Figure 5).

## Discussion

Formation of the herpesvirus capsid is the first step in viral morphogenesis. The capsid of HSV-1 is found in the mature virions and in the nuclei of infected cells from which they originate. There are three distinct types of capsid, A-, B- and C-capsids, in infected cells [6]. The B-capsids of HSV-1 contain a large amount of the scaffolding protein (the product of the *UL26.5 *gene), and smaller amounts of VP24 and VP21, the products of the *UL26 *gene. The *UL26 *and *UL26.5 *genes are expressed as 3'-coterminal transcripts, and the promoter for the *UL26.5 *gene is located within the coding region of the *UL26 *gene in HSV-1 [27-29]. The *UL26 *gene encodes a self-cleaved protease that generates the capsid proteins VP21 and VP24 [30]. Cleavage of the UL26 and UL26.5 proteins is not required for capsid assembly, but the cleavage event is essential for DNA encapsidation [31]. The *UL26.5 *gene encodes the scaffold protein VP22a [27], which has been linked to a family of proteins named infected-cell protein 35 (ICP35). The VP22a is the most abundant protein found in the B-capsid and may form the scaffold of the HSV-1 B-capsid [32]. The B-capsid is similar to the capsid found in infectious HSV-1 particles, except that it lacks DNA. The cavity of the B-capsid is occupied instead by a proteinaceous core that is removed when DNA enters [32]. The UL26 proteinase cleaves the products of the *UL26.5 *gene, and itself, at a site 25 amino acids from the C terminus of its product [27]. DEV is an unassigned member in the family *Herpesviridae *and, to date, there has been no report of the structure and function of proteins UL26 and UL26.5 in DEV. It has been reported that the genes *UL26 *and *UL26.5 *in DEV are similar to their homologues in other alphaherpesviruses [20]. It has been speculated that they have similar functions in viral infection and replication to those of HSV-1 and other alphaherpesviruses.

**Figure 6:**
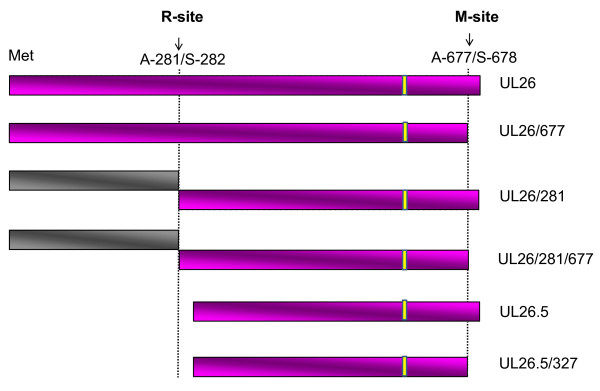
**Schematic representation of UL26 and UL26.5 in the DEV Clone-03 strain**. The location of the R and M cleavage sites are indicated by the arrows and the dashed lines indicate the cleavage sites. The results were based on the alignment of the structure of UL26 in DEV and HSV-1 [36,39]. The sequence of the epitope is indicated by the yellow bars. Each of the proteins is designated by UL26 or UL26.5 plus the sites of cleavage. The pink bars represent the amino acid sequence that contains the epitope defined by mAb 1C8. The gray bars represent the proteins that were not recognized by mAb 1C8.

In this study, we expressed the C-terminus of DEV UL26 and immunized mice with both recombinant protein UL26c and the DEV particles, using a prime-boost protocol, and one mAb (1C8) was generated by cell fusion. Interestingly, six bands were detected in DEV-infected CEFs in western blotting analysis using mAb 1C8 in this study (Figure 1B). These may have originated from cleavage by the products of the *UL26 *gene. Figure 6 shows the proteins that are predicted to originate from the products of the DEV *UL26 *and *UL26.5 *by the alignment of the homologous in HSV-1. The DEV *UL26 *gene encodes a deduced protein of 707 amino acids [20]. Comparison of the amino acids in DEV UL26 with the homologous in HSV-1 showed that DEV UL26 contains two sites. The release site (R) was at amino acids 281 and 282, and the maturational site (M) was at amino acids 677 and 678. Self-cleavage by UL26 may yield the proteolytically active VP24-like protein and VP21-like protein (Figure 6) [30], which are approximately 31 kD and 43 kD, respectively. The *UL26.5 *gene is in frame with *UL26*; therefore, the UL26.5-encoded proteins possess the same M site as the UL26 protein. Consequently, we hypothesized that the six bands detected using western blotting in this study may indicate the six products of UL26 and UL26.5.

A series of 17 fragments that spanned the UL26c protein were expressed with a GST tag in this study, and used to screen for the minimal epitope recognized by mAb 1C8 using western blotting and ELISA. It was demonstrated that the minimal sequence of the epitope defined by mAb 1C8 appeared to be ^520^IYYPGE^525^, because any deletion of residues from either end of ^520^IYYPGE^525 ^destroyed the ability of mAb 1C8 to bind. Comparative analysis of the amino acid sequences of the identified epitope with those of another 14 alphaherpesviruses revealed that the C-terminus of the linear B-cell epitope ^521^YYPGE^525 ^is conserved among the selected alphaherpesviruses, except PRV, VZV and ILTV. It has been reported previously that the motif YYPGE is conserved in the scaffold proteins of alphaherpesviruses [33]. It was reported that deletion of a 9-amino acid motif from the HSV-1 UL26.5, comprising amino acids 143 to 151, which contained the sequence YYPGE, did not affect the formation of capsids *in vitro *but had a specific effect on incorporation of the portal. This indicated that this deletion had blocked DNA packing but had not interfered with the assembly of B-capsids [33,34]. Therefore, the 9-amino acid motif that contains YYPGE is required for the formation of portal-containing capsids in HSV-1-infected cells, and it is essential for the production of infectious virus [34]. However, in DEV, the function of the motif needs further investigation.

The result of the *in vitro *neutralization test showed that mAb 1C8 could not neutralize the infectivity of DEV. The absence of neutralizing activity against DEV might indicate that this region has low immunogenicity or, more probably, that this region is not exposed on the surface of the virion. Indeed, the products of the HSV-1 *UL26 *gene are components of viral capsids, which are located inside of the viral tegument and envelope, while the products of the *UL26.5 *gene are components of B-capsids [28]. The products encoded by the two genes show predominantly nuclear localization within infected cells [35]. The B-capsids are accumulated in the nucleus prior to viral DNA encapsidation [36] and the predominant component of B-capsids is the product of the *UL26.5 *gene, which also contains the sequences of the epitope defined by 1C8.

The mAb 1C8 and its epitope, which was defined in this study, may prove to be very useful tools for the development of immunity-based therapeutic and diagnostic techniques for DEV, although the mAb lacked neutralizing ability.

## Conclusion

In this study, we generated a mAb, 1C8, and identified a novel linear B-cell epitope on the DEV UL26 and UL26.5 proteins using this mAb. The identified epitope and mAb 1C8 may increase our understanding of the function and location of the UL26 and UL26.5 proteins in DEV. It will also be a potential tool for the design of diagnostic reagents for DEV.

## Methods

### Cells and virus strain

The SP2/0 cells were maintained in Dulbecco's Modified Eagle's Medium (DMEM) (Invitrogen, USA) supplemented with 10% fetal bovine serum (FBS) (Invitrogen, USA) and 1% penicillin-streptomycin in a humidified 5% CO_2 _atmosphere at 37°C. Chicken embryo fibroblasts (CEF) were prepared from 9- to 11-day-old specific-pathogen-free (SPF) embryonated eggs according to standard procedures. The DEV Clone-03 strain was purified from a commercial Chinese DEV vaccine using the plaque assay, as described previously [19]. The virus was propagated in CEF in DMEM with 8% FBS and harvested when the cytopathic effect (CPE) reached 80%. After three freeze-thaw cycles, cell lysates were confirmed primarily by electron microscopy (JEM-1200 EX, Japan) and polymerase chain reaction (PCR) [20].

### Gene cloning, construction of recombinant expression vectors and expression of fusion proteins

Given that it is difficult to induce prokaryotic expression of the full *UL26 *gene, we expressed a fragment of 1083 bp at the C-terminus of the DEV *UL26 *gene (*UL26c*) by amplifying the gene fragment from DEV Clone-03. The sequences and locations of the primers used for amplification and expression of the gene in this study are shown in Table 1.

**Table 1:** Sequences of the primers used in this study

Fragments	**Primer sequences (5' - 3')**^**a**^		**Position in UL26 gene**^**b**^	Size of amplicon (bp)
			
	Sense	Negative sense		
UL26c	GGATCC**ATG**CAATCTACTATGACG	GTCGAC**TCA**ACATCTATTACACATCA	1042-2124	1083
F1	GGATCC**ATG**CAATCTACTATGACG	GTCGAC**TTA**CAGCTGCCCTCCCTGGAC	1042-1347	306
F2	GGATCC**ATG**TATGGACAGCCTGTTTAT	GTCGAC**TTA**AGCTAATGGTCCAGTAGA	1294-1731	438
F3	GGATCC**ATG**CCTACTGGACAAGGTAAC	GTCGAC**TCA**ACATCTATTACACATCA	1681-2124	444
F2-1	GGATCC**ATG**TATGGACAGCCTGTTTAT	CTTTGGTCGAC**TTA**ATCTCCAGATTCGACGGC	1294-1455	162
F2-2	TGCAGGGATCC**ATG**GCAATTGCTGCAGATAGG	CTTTGGTCGAC**TTA**TTCCCCCGGATAATAGAT	1411-1575	165
F2-3	TGCAGGGATCC**ATG**AATGACGACCAGTTAGAT	GTCGAC**TTA**AGCTAATGGTCCAGTAGA	1531-1731	201
F4	TGCAGGGATCC**ATG**AATGACGACCAGTTAGAT	CTTTGGTCGAC**TTA**TTCCCCCGGATAATAGAT	1531-1575	45
F5	TGCAGGGATCC**ATG**GACGACCAGTTAGATGGT	CTTTGGTCGAC**TTA**TTCCCCCGGATAATAGAT	1534-1575	42
F6	TGCAGGGATCC**ATG**CAGTTAGATGGTGACAAT	CTTTGGTCGAC**TTA**TTCCCCCGGATAATAGAT	1540-1575	36
F7	TGCAGGGATCC**ATG**TTAGATGGTGACAATATC	CTTTGGTCGAC**TTA**TTCCCCCGGATAATAGAT	1543-1575	33
F8	TGCAGGGATCC**ATG**GATGGTGACAATATCTAT	CTTTGGTCGAC**TTA**TTCCCCCGGATAATAGAT	1546-1575	30
F9	TGCAGGGATCC**ATG**GGTGACAATATCTATTAT	CTTTGGTCGAC**TTA**TTCCCCCGGATAATAGAT	1549-1575	27
F10	TGCAGGGATCC**ATG**GACAATATCTATTATCCG	CTTTGGTCGAC**TTA**TTCCCCCGGATAATAGAT	1552-1575	24
F11	TGCAGGGATCC**ATG**AATATCTATTATCCGGGG	CTTTGGTCGAC**TTA**TTCCCCCGGATAATAGAT	1555-1575	21
F12	TGCAGGGATCC**ATG**ATCTATTATCCGGGGGAA	CTTTGGTCGAC**TTA**TTCCCCCGGATAATAGAT	1558-1575	18
F13	GATCC**ATG**ATCTATTATCCGGGG**TAA**G	TCGAC**TTA**CCCCGGATAATAGAT**CAT**G	1558-1572	15
F14	GATCC**ATG**TATTATCCGGGGGAA**TAA**G	TCGAC**TTA**TTCCCCCGGATAATA**CAT**G	1561-1575	15

^a ^The introduced restriction enzyme sites (*Bam *HI and *Sal *I) in each primer are underlined. The stop codon, TCA, of the DEV *UL26 *gene and the introduced start and stop codons in the primers of the UL26 fragments are shown in bold, respectively.

^b ^The nucleotide positions correspond to those of the DEV *UL26 *gene, GenBank accession no. EF203709.

After construction, each recombinant expression construct was transformed into *E. coli *BL21 (DE3) (Novagen, Gibbstown, NJ, USA). A series of fusion proteins with the expected molecular weights were induced by IPTG and stained with Coomassie blue after SDS-PAGE as described previously [37]. For preparation of purified proteins, inclusion body proteins were separated by SDS-PAGE, the proteins of interest were excised, and the gel slices were crushed and added to an appropriate volume of sterilized PBS. The extracted proteins were used for western blotting and ELISA.

### Generation of mAbs against the DEV UL26 protein

Three 8-week-old BALB/c mice were primed subcutaneously with DEV virus particles mixed with an equal volume of Freund's complete adjuvant (Sigma, USA), followed by two boosts of immunization with the recombinant protein UL26c. The protocols used for the preparation of mAbs and ascitic fluid have been described previously [37,38]. All hybridomas were cloned by at least three rounds of limiting dilution. The class and subclass of the mAb produced were determined using an SBA Clonotyping™ System/HRP kit (Southern Biotechnology Associates, Birmingham, USA).

### SDS-PAGE and western blotting

The specificity and reactivity of the mAb 1C8 were also determined by western blotting using recombinant UL26c protein and CEF infected by DEV, respectively. Purified recombinant UL26c protein, truncated proteins and DEV in CEF were separated by denaturing SDS-PAGE. For western blotting, all the proteins and the virus were transferred onto nitrocellulose membranes, and detected as described previously [37]. Purified recombinant GST (rGST) protein and the CEF were used as negative antigen controls.

### Indirect ELISA

The reactivity of the mAb with different truncated recombinant UL26 proteins was determined further by ELISA as described previously [37]. Briefly, the purified recombinant proteins were used as coating antigens, applied at 10 μg/well in 0.1 M carbonate buffer (pH 9.6) at 4°C for 12 h, and blocked with 0.5% BSA at 37°C for 1 h. After washing three times with PBST, 100 μl of mAb were added to the wells and incubated at 37°C for 1 h. The plates were washed three times and incubated with HRP-conjugated sheep anti-mouse secondary antibody at 37°C for 1 h. The color was developed and the reaction terminated, and the absorbance was measured at 490 nm. All assays were performed in triplicate.

### In vitro neutralization test

The mAb 1C8 was tested for the presence of DEV-neutralizing antibodies. The CEF lysates containing DEV were mixed with ascites fluid containing mAb 1C8 and incubated at 37°C for 1 h; unrelated ascites fluid and PBS, used as negative controls, were treated in the same way. The mixture was added to the prepared CEF and incubated at 37°C. After 2 h of incubation, the mixture was removed and the cells were overlaid with 1% low-melting-point agarose containing 8% FBS. At 72 h post-incubation, the cells were overlaid again with 1% low-melting-point agarose containing 0.1% Ponceau. After further incubation at 37°C for 24 h, the plaques were counted and compared.

### Detection of the reactivity of the epitope defined by mAb 1C8

To investigate whether the peptides could be recognized by anti-DEV antibody, the epitope peptide F12 was purified and used as antigen to coat ELISA plates (10 μg/well) to react with mouse anti-DEV antibody and mouse sera, respectively. Purified rGST was used as the negative control for F12. In addition, the purified F12 and rGST were also used to detect the reactivity of the epitope peptide by western blotting.

### Homologous analysis of the sequence of the epitope defined by mAb 1C8

The mAb 1C8-defined epitope sequences and flanking sequences of DEV were compared with those of 14 other selected herpesviruses of the *Alphaherpesvirinae *using the MEGALIGN program in Lasergene (DNAStar) with CLUSTAL W multiple alignments, as described previously [23]. The 14 herpesviruses used in this study were as follows: equine herpesvirus 1 (EHV-1) (NC_001491), equine herpesvirus 4 (EHV-4) (AF030027), pseudorabies virus (PRV) (BK001744), varicella-zoster virus (VZV) (NC_001348), bovine herpesvirus 1 (BHV-1) (AJ004801), bovine herpesvirus 5 (BHV-5) (AY261359), herpes simplex virus type 1 (HSV-1) (X14112), herpes simplex virus type 2 (HSV-2) (NC_001798), cercopithecine herpesvirus 1 (CeHV-1) (NC_004812), cercopithecine herpesvirus 2 (CeHV-2) (NC_006560), Marek's disease virus 1 (MDV-1) (AF243438), Marek's disease virus 2 (MDV-2) (AB049735), turkey herpesvirus (HVT) (AF291866), and infectious laryngotracheitis virus (ILTV) (NC_006623).

## Competing interests

The authors declare that they have no competing interests.

## Authors' contributions

XL, SL and XK designed research; XL, ZH, YS and DY performed research; XL, SL, XK, HL and YW analyzed data; and XL, SL and XK wrote the paper. All authors read and approved the final manuscript.
